# Income-related inequalities in unmet dental care needs in Spain: traces left by the Great Recession

**DOI:** 10.1186/s12939-020-01317-x

**Published:** 2020-11-12

**Authors:** Rosa M. Urbanos-Garrido

**Affiliations:** Department of Applied Economics, Public Economics and Political Economy, School of Economics & Business, University Complutense of Madrid, Campus de Somosaguas, 28223 Pozuelo de Alarcón Madrid, Spain

**Keywords:** Unmet dental care needs, Income-related inequality, Spain, Great Recession, H51, I14, I18

## Abstract

**Background:**

Dental health is an important component of general health. Socioeconomic inequalities in unmet dental care needs have been identified in the literature, but some knowledge gaps persist. This paper tries to identify the determinants of income-related inequality in unmet need for dental care and the reasons for its recent evolution in Spain, and it inquires about the traces left by the Great Recession.

**Methods:**

Data from the EU-SILC forming a decade (2007–2017) were used. Income-related inequalities for three years were measured by calculating corrected concentration indices (CCI), which were further decomposed in order to compute the contribution of different factors to inequality. An Oaxaca-type decomposition approach was also used to analyze the origin of changes over time. Men and women were analyzed separately.

**Results:**

Pro-rich inequality in unmet dental care needs significantly increased over time (CCI 2007: − 0.0272 and − 0.0334 for males and females, respectively; CCI 2017: − 0.0704 and − 0.0776; *p* < 0.001). Inequality showed a clear “pro-cycle” pattern, growing during the Great Recession and starting to decrease just after the economic recovery began. Gender differences only were significant for 2009 (*p* = 0.004) and 2014 (*p* = 0.063). Income was the main determinant of inequality and of its variation along time -particularly for women-, followed by far by unemployment –particularly for men-; the contributions of both were mainly due to changes in elasticites.

**Conclusions:**

The Great Recession left its trace in form of a higher inequality in the access to dental care. Also, unmet need for dental care, as well as its inequality, became more sensitive to the ability to pay and to unemployment along recent years. To broaden public coverage of dental care for vulnerable groups, such as low-income/unemployed people with high oral health needs, would help to prevent further growth of inequality.

**Supplementary Information:**

The online version contains supplementary material available at 10.1186/s12939-020-01317-x.

## Background

Dental health is, undoubtedly, an important component of general health [1–3]. Although its relevance is widely recognized, public coverage of dental care is quite limited in most Mediterranean European countries, where oral system of health care is mainly private [4]. This is the case of Spain, where only extractions and emergency care for adults are publicly provided [5]. According to the System of Health Accounts, out-of-pocket expenditure accounted in 2017 for 98.8% of the total dental expenditure in Spain, which in turn represents 28.6% of total households’ health expenditure for the same year [6]. While the government and compulsory insurance spending as proportion of total spending in dental care reaches 29% for the OECD as a whole, Spain barely shows 2%, only ahead of Greece [7].

In this context, the presence of socioeconomic barriers of access to dental care is expected [8]. According to Eurostat data, more than 90% of people reporting unmet dental care needs in Spain in 2018 declared that the main reason for not receiving dental care when needed was that they could not afford the cost [9]. The variability in the financing of oral health care partially explains differences in the non-use of dental care across countries [10, 11]. In 2018, the percentage of population reporting unmet dental care needs in Spain reached 5.4%, higher than the EU and the euro area averages (4.1 and 3.9%, respectively) [9].

The Great Recession, officially starting at 2008, hit Spanish society hard in the context of the European Union, and represented a serious economic shock for Spanish households. The unemployment rate reached 26.1% in 2013, more than doubling the EU (10.8%) and the eurozone averages (12.0%) and quite near the maximum corresponding to Greece (27.5%) [12]. Furthermore, the AROPE (At Risk Of Poverty or Social Exclusion) rate rose up to 29.2% in 2014, only behind Greece (36.0%) and Latvia (32.7%) in the euro area [13]. The economic crisis also had a serious impact in terms of inequality. The Gini index reached 34.7 (in a scale from 0 to 100) in 2014, almost five percentage points over the EU average (31) in the same year [14]. This scenario constitutes a fertile ground for socio-economic inequalities in the access to dental care, but also for the rest of health care services, since severe measures were applied by the Spanish government to control public health spending, including restrictions to universal healthcare coverage. Several studies have shown that socio-economic inequalities in the access/use of health care services tended to increase during the Great Recession, not only in Spain [15–17], but also in other European countries [18].

Substantial empirical research has previously addressed socioeconomic determinants of access and use of dental care for adults [19–26] and, more specifically, of unmet dental care needs [10, 11, 27–36]. However, most studies focused on particularly vulnerable groups, such as the elderly [30, 34], homeless [28], HIV/AIDS patients [27], low-income [29] or disabled adults [33, 36]. Also, the effect of the Great Recession on unmet needs and its determinants has received little attention [11, 32, 36]. In Spain, one of the European countries which suffered most the economic crisis, Calzón et al. (2015) analyzed socioeconomic inequalities in unmet dental care needs for working-age population by using the Spanish sample of the EU-SILC from the years 2007 and 2011, in order to assess the impact of the crisis [32]. Based on that paper, the present research tries to cover some knowledge gaps by, firstly, extending the analyzed period, in order to inquire about the traces left by the Great Recession. Secondly, it tries to identify the determinants of income-related inequality in unmet need for dental care and the reasons for its evolution along recent years. Finally, the paper adopts a broader scope compared to precedent studies by including elderly people in the analysis. This research may contribute to know how sensitive inequalities in unmet needs for dental care are to fluctuations in the economic cycle. Also, it may help to identify the underlying inequality factors in access to dental care, which will be useful to policy makers to address this problem.

**Fig. 1:**
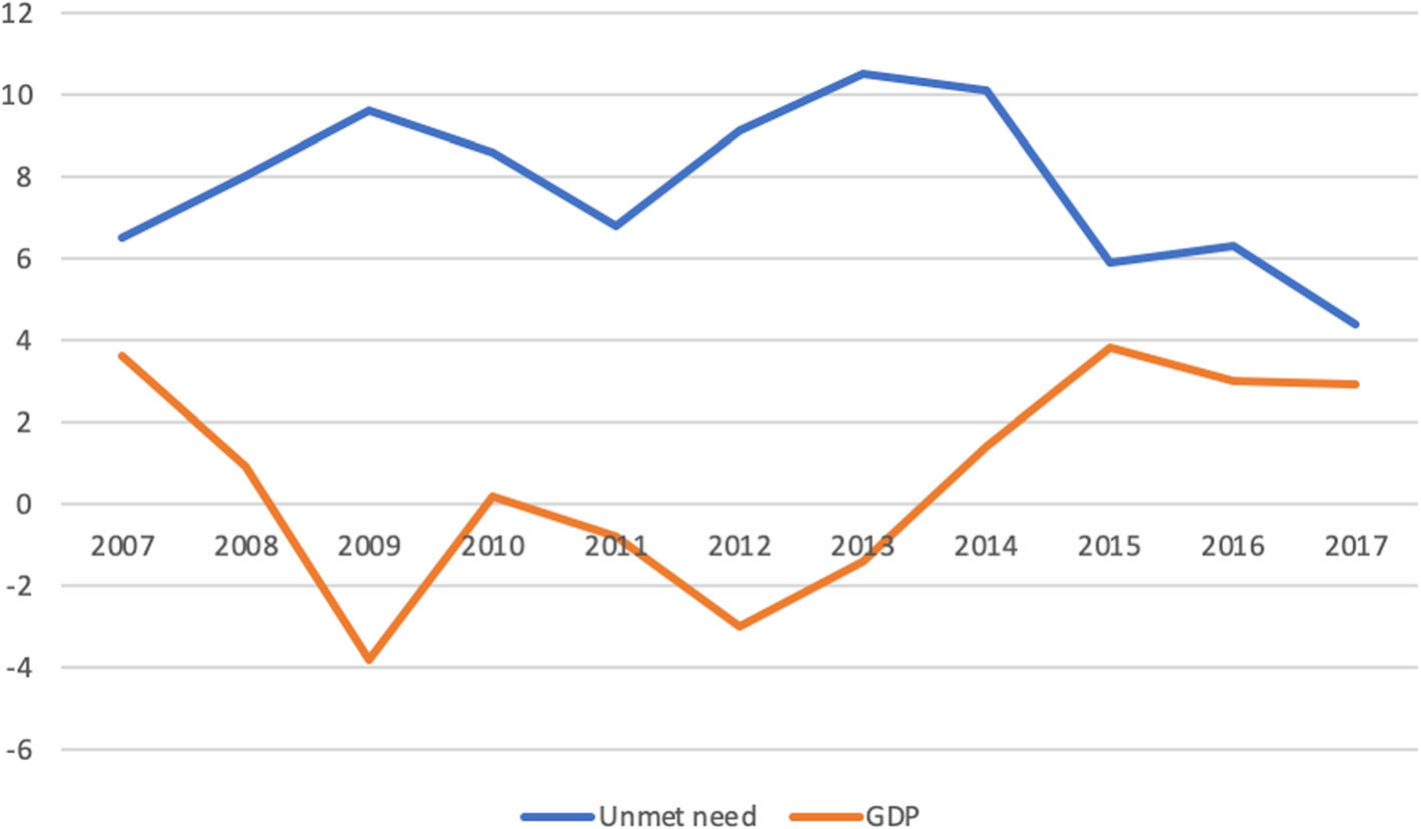
Evolution of GDP and unmet needs for dental care in Spain 2007–2017 (%). Source: own elaboration from Eurostat database and INE

With these aims, conventional methods of measuring income-related inequalities (IRI) were applied to the decade 2007–2017. Also, IRI and its evolution were further decomposed for three different years. The first selected year (2007) represents the pre-crisis time, when the Spanish GDP was growing over 3% [37]. As it may be observed in Fig. 1, the GDP growth registered a sharp fall a year after and, at the same time, unmet need for dental care began to rise. The second year (2012), that divides the decade in two halves, registered the hardest fall of the GDP (3%) after 2009 (with a decrease reaching 3.8%). The trend followed by the Spanish GDP started to change from that year on. Moreover, in 2012 the Spanish Government introduced a package of reforms in order to contain public healthcare expenditure through the Royal Decree-Law 16/2012, including the revocation of full right to public health care coverage for undocumented migrants and some other groups [38]. The selected post-recession year (2017) corresponds to a new period of economic growth (with GDP growing at 3%) and represents a turning point for unmet dental care need, which began to rise from that moment. According to Eurostat, the proportion of people declaring unmet need reached 4.4% in 2017, 5.4% in 2018 and 6.1% in 2019 [9]. At the same time, the Spanish GDP growth began to visibly slow down, with rates reaching 2.4 and 2% in 2018 and 2019, respectively [37].

In order to incorporate the gender perspective, the analysis was done separately by men and women.

## Material and methods

### Material

Data from the Spanish sample of the Statistics on Income and Living Conditions (EU-SILC) for the whole period 2007–2017 were used. EU-SILC provides data on income and living conditions in the European Union, which are collected at the household level. It also collects personal information about health, including unmet needs for medical treatments and dental care, and other characteristics such as labour status or education, which refer to individuals aged 16 and over. Since it provides accurate information about disposable income, this database is particularly adequate to analyze income-related inequality of unmet needs. The EU-SILC is set up as a rotating panel where the sample is completely renewed every 4 years. Since the 3 years selected to compute the contribution of different factors to inequality are separated by a 5-year gap, they form a repeated cross-sectional dataset.

### Methods

Concentration indices, which have been widely used in the measurement of income-related inequality and have also been applied to dental care [22, 23, 26, 31, 32] were employed in this study. Given that unmet need for dental care is represented by a binary variable, the corrected concentration index (CCI) proposed by Erreygers (2009) [39], which represents an absolute index of inequality [40], was used.

This index can be expressed as follows:

1 $$ CCI=\frac{8}{n^2}{\sum}_{i=1}^n{y}_i{r}_i $$

where *y* is the variable of interest, and *r*_*i*_ is the cumulative percentage that each individual *i* = 1, …, *n* represents over the total population, after being ranked by income. Positive (negative) values of the index mean that inequality favors the better-off (worse-off), whilst if it is not significantly different from zero it may be concluded that there is no income-related inequality in the distribution of the variable *y*. When the dependent variable links linearly and additively to a set of *k* explanatory variables *x*, it may be expressed as:

2 $$ y=\alpha +{\sum}_k{\beta}_{k\kern0.5em }{\overline{x}}_k+e, $$

where *e* is the error term. Following the decomposition method by Wagstaff et al. (2003) [41], the CCI may be written as a weighted sum of the partial corrected concentration indices for the explanatory factors of inequality, being the weight the elasticity of *y* with respect to *x*_*k*_:

3 $$ CCI=\sum k\frac{\left({\beta}_k{\overline{x}}_k\right)}{\overline{y}}{CCI}_k+4\bullet {GCI}_e $$

where GCI_e_ is the generalized concentration index for the error term. As the outcome variable is dichotomous, non-linear models are preferred. In our case, probit models were used to carry out the estimates. Then, the decomposition of the corrected concentration index is only possible if some linear approximation is made to the non-linear model. This can be done by substituting *β*_*k*_ coefficients by the partial effects ($$ \raisebox{1ex}{$ dy$}\!\left/ \!\raisebox{-1ex}{$d{x}_k$}\right.\Big) $$ evaluated at sample means $$ \left({\beta}_k^m\right) $$, in eqs. (2)–(3).

Moreover, changes in income-related inequality over time may be expressed as (4):

4 $$ \Delta  CCI={CCI}_t-{CCI}_1=\frac{8}{n^2}\left({\sum}_{i=1}^n{y}_{it}{r}_{it}-{\sum}_{i=1}^n{y}_{i1}{r}_{i1}\right) $$

which can be further disentangled by using an Oaxaca-type decomposition [41], such that variation of inequality can be explained by changes in elasticities and by changes in *CCI*_*k*_:

5 $$ \Delta  CCI={\sum}_k\left(\frac{\beta_{k,t}{\overline{x}}_{k,t}}{{\overline{y}}_t}\right)\left({CCI}_{k,t}-{CCI}_{k,t-1}\right)+{\sum}_k{CCI}_{k,t-1}\left(\frac{\beta_{k,t}{\overline{x}}_{k,t}}{{\overline{y}}_t}-\frac{\beta_{k,t-1}{\overline{x}}_{k,t-1}}{{\overline{y}}_{t-1}}\right)+4\bullet \Delta  {GC}_e $$

or, given that the Oaxaca decomposition is not unique, alternatively by:

6 $$ \Delta  CCI={\sum}_k\left(\frac{\beta_{k,t-1}{\overline{x}}_{k,t-1}}{{\overline{y}}_{t-1}}\right)\left({CCI}_{k,t}-{CCI}_{k,t-1}\right)+{\sum}_k{CCI}_{k,t}\left(\frac{\beta_{k,t}{\overline{x}}_{k,t}}{{\overline{y}}_t}-\frac{\beta_{k,t-1}{\overline{x}}_{k,t-1}}{{\overline{y}}_{t-1}}\right)+4\bullet \Delta  {GC}_e $$

### Definition of variables

The variable of interest, unmet need for dental care, is a dummy that takes value of 1 if the individual declares that, at least once in the latest year, he/she has not received dental care when needed, and 0 otherwise.

The selection of independent variables tries to include all those factors related to the underlying reasons for unmet need which are reported in the EU-SILC. Age, nationality and marital status represent socio-demographic characteristics, and chronicity is used as a proxy of health status. The choice of the age groups (16–34, 35–64 and over 64) fits well with the different economic stages for Spaniards: young adults economically dependent from their parents, independent adults in working age and retired population. According to Eurostat, in 2018 the share of Spanish young adults (aged 18–34) living with their parents reached 62.8% [42]. Moreover, the regional density of professionals per 100,000 inhabitants [43] was added in order to test the relevance of supply as a possible determinant of unmet need. Finally, educational level, labor status and household income represent the socioeconomic position of individuals.

Household income is defined as the annual net household income in the year preceding the interview. Index prices supplied by the Spanish Statistical Office were used to express income in constant values of 2017. The modified OECD equivalence scale was employed to calculate equivalent income. Following Coveney et al. (2016), observations with negative incomes were removed, which only represent 0.18, 0.37 and 0.14% of the whole sample in 2007, 2012 and 2017, respectively [44].

The analysis was performed using StataSE 13©. The CCI were calculated by using the conindexcommand [45]. Individual cross-sectional weights were applied to make the sample representative of the whole population. The final sample consisted of 26,772 observations in 2007 (12,935 men and 13,837 women), 26,311 in 2012 (12,818 men and 13,493 women) and 28,932 in 2017 (13,821 men and 15,111 women). Table 1 shows the definition of the variables and descriptive statistics of the sample.

**Table 1:** Definition of variables and descriptive statistics of the sample

		Mean 2007 (Std. Dev.)		Mean 2012 (Std. Dev.)		Mean 2017 (Std. Dev.)	
Variables	Definition	Men (*n* = 12,935)	Women (*n* = 13,837)	Men (*n* = 12,818)	Women (*n* = 13,493)	Men (*n* = 13,821)	Women (*n* = 15,111)
Unmet need	1 if at least once in the latest year dental care was not received when needed; 0 otherwise	6.9%	6.1%	8.8%	9.4%	4.1%	4.7%
age16–34	1 if aged from 16 to 34; 0 otherwise (reference category)	35.9%	33.6%	30.5%	29.0%	25.2%	23.5%
age35–64	1 if aged from 35 to 64; 0 otherwise	49.7%	48.6%	53.2%	51.9%	54.8%	52.1%
age65+	1 if aged 65 or over; 0 otherwise	14.4%	17.9%	16.3%	19.1%	20.0%	24.4%
Spanish	1 if Spanish nationality; 0 otherwise	91.2%	90.4%	89.3%	89.3%	92.1%	91.9%
single	1 if single; 0 otherwise (reference category)	38.1%	30.3%	36.9%	28.8%	36.2%	27.6%
married	1 if married or living in couple; 0 otherwise	55.9%	54.4%	56.8%	54.6%	55.7%	52.6%
separated	1 if separated or divorced; 0 otherwise	3.5%	4.9%	3.7%	6.0%	4.7%	7.0%
widowed	1 if widowed; 0 otherwise	2.6%	10.4%	2.6%	10.6%	3.4%	12.8%
chronic	1 if any chronic condition is reported; 0 otherwise	22.3%	24.1%	22.8%	25.5%	26.4%	30.7%
dentxinhab	Number of dentists per 100.000 inhabitants	53.764 (13.273)	54.043 (13.337)	66.752 (18.489)	67.309 (18.779)	78.276 (20.319)	78.784 (20.581)
primary_educ	1 if primary education or less; 0 otherwise (reference category)	27.9%	31.6%	23.1%	26.3%	21.0%	25.6%
lowsec_educ	1 if lower secondary education; 0 otherwise	25.1%	21.9%	27.9%	23.6%	27.4%	23.5%
uppersec_educ	1 if upper secondary or post-secondary non-tertiary education; 0 otherwise	22.3%	22.8%	22.9%	22.7%	23.6%	21.4%
tertiary_educ	1 if tertiary education; 0 otherwise	24.7%	23.7%	26.1%	27.4%	28.0%	29.5%
working	1 if working; 0 otherwise (reference category)	65.8%	45.4%	51.9%	40.4%	54.5%	42.8%
unemployed	1 if unemployed; 0 otherwise	5.9%	6.7%	17.6%	15.2%	11.4%	11.6%
inactive	1 if inactive; 0 otherwise	28.3%	47.9%	30.5%	44.4%	34.1%	45.6%
Ln_eqincome	Log of the equivalent household income (euros 2017)	9.498 (0.641)	9.448 (0.666)	9.531 (0.719)	9.515 (0.723)	9.495 (0.728)	9.463 (0.748)

## Results

According to Table 1, the evolution of unmet need for dental care in Spain along the decade 2007–2017 has been globally positive. Although the proportion of population reporting unmet needs significantly increased from the pre-recession year to 2012, particularly among women, it tended to decline afterwards below the starting point. Changes in the percentages corresponding to the age dummies clearly reflect the growing ageing process in Spain, whilst differences between men and women reflect the higher life expectancy for females. However, the chronicity burden, that remained constant during the first half of the analyzed period and tended to increase during the second half, is systematically higher for women.

Moreover, the ratio of dentists per 100,000 inhabitants shows how the supply of services steadily grew in Spain along the decade. Also, data on Table 1 indicate how educational level tended to improve, with a growing proportion of population with university studies. The biggest changes along the period took place in the job market. Unemployment steeply grew from 2007 to 2012, more than doubling the starting level (and almost tripling it for males). Although unemployment rate tended to decrease after 2012, in 2017 –and after 3 years of economic growth- it was still notably higher than it was before the Great Recession. Also, the proportion of inactive men (retired, handicapped, students and houseworkers) slightly tended to increase since 2007. Conversely, the rate of inactive women showed a decline in the first half of the considered decade.

**Fig. 2:**
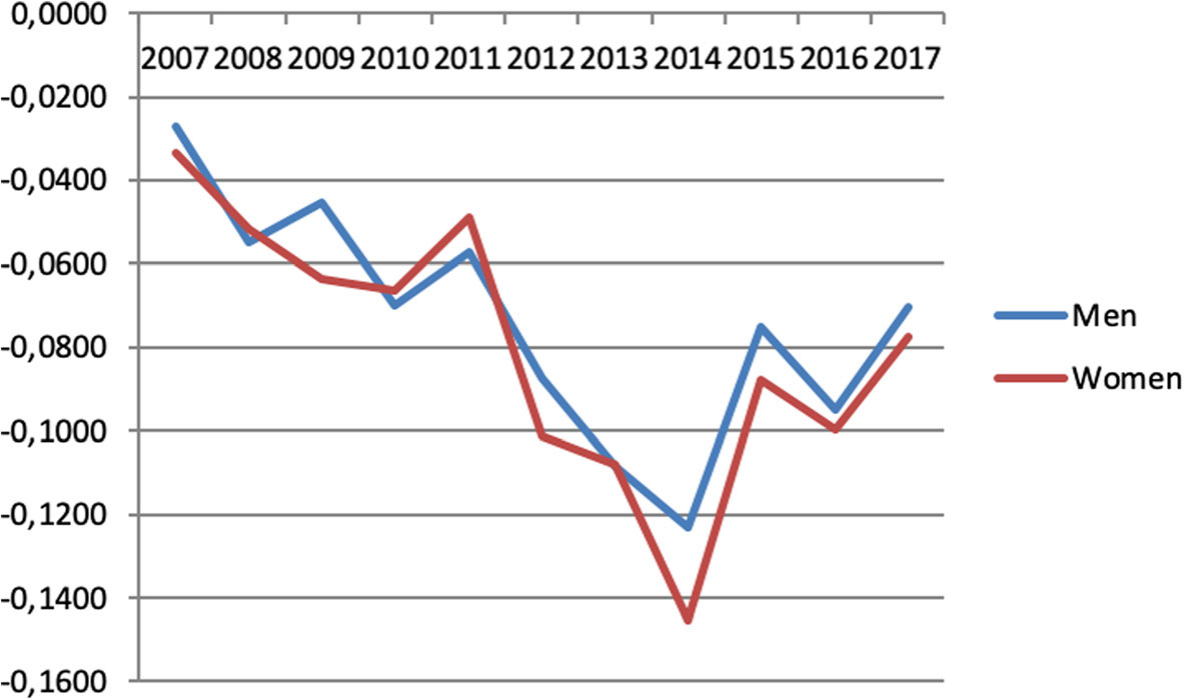
Evolution of income-related inequalities (corrected concentration indices) in unmet dental need in Spain (2007–2017). Source: own elaboration from EU-SILC

Figure 2 shows that income-related inequality in unmet dental care needs systematically favoured the better-off along the whole period. All the CCIs are negative and statistically significant at 99% level (*p* < 0.001). Inequality followed the same pattern for men and women, being gender differences significant only for 2009 (*p* = 0.004) and 2014 (*p* = 0.063). In general terms, inequality showed a “pro-cycle” pattern, since it tended to grow during the years of economic crisis and started to decrease just after the economic recovery began. The CCIs reached in 2007 – 0.0272 and − 0.0334 for males and females, respectively. In the middle of the ten-year period (2012), indices increased up to − 0.0875 for men and to − 0.1012 for women, and kept on growing until 2014. Finally, inequality notably decreased from 2014 to the end of the considered decade, although without achieving the pre-crisis levels: in 2017 the Erreygers’ indices reached − 0.0704 and − 0.0776 for men and women, respectively.

Tables 2 & 3 show the contributions to inequality in unmet needs for dental care for each analyzed year for men and women, respectively. The first column for every year reports the estimated partial effects from the probit model. The second and third column show the elasticity of unmet need for each regressor and its CCI, respectively. Moreover, the fourth column reports the absolute contribution of each variable to overall inequality, which is the product of the elasticity and the partial Erreygers’ index. The fifth column reports the percentage contribution, which is obtained by dividing the absolute contribution by the overall income-related inequality. A positive (negative) percentage contribution implies that, if inequality in unmet need for dental care was determined by that variable alone, it would favor the better-off (worse-off). Finally, the last column of the table displays the absolute change in contributions for the whole period (2007–2017).

**Table 2:** Contributions to inequality in unmet need for dental care in 2007, 2012 and 2017, and decomposition of total change. Men

	2007					2012				
Variables	Partial effect	Elasticity	CCI	Contribution (1)	% Contribution	Partial effect	Elasticity	CCI	Contribution (2)	% Contribution
age35–64	0.0180*	0.1303	0.0054	0.0007	−2.6%	0.0211*	0.1278	0.0068	0.0009	−1.0%
age65+	− 0.0038	− 0.0079	− 0.0460	0.0004	− 1.3%	0.0040	0.0074	0.0064	0.0000	−0.1%
Spanish	− 0.0020	− 0.0264	0.0057	− 0.0001	0.5%	− 0.0419***	− 0.4245	0.0169	− 0.0072	8.2%
married	0.0070	0.0568	−0.0038	−0.0002	0.8%	0.0129	0.0833	0.0052	0.0004	−0.5%
separated	0.0381	0.0191	0.0133	0.0003	−0.9%	0.0316*	0.0134	−0.0172	− 0.0002	0.3%
widowed	−0.0148	− 0.0056	− 0.0147	0.0001	− 0.3%	0.0445*	0.0134	0.0280	0.0004	−0.4%
chronic	0.0396***	0.1284	−0.0265	− 0.0034	12.5%	0.0555***	0.1438	− 0.0163	−0.0023	2.7%
dentxinhab	−0.0002	− 0.1447	0.0067	− 0.0010	3.5%	0.0001	0.0881	0.0097	0.0009	−1.0%
lowsec_educ	−0.0102	− 0.0372	− 0.0290	0.0011	−4.0%	0.0032	0.0102	−0.0527	− 0.0005	0.6%
uppersec_educ	−0.0206**	− 0.0668	0.0163	− 0.0011	4.0%	− 0.0073	− 0.0188	0.0107	− 0.0002	0.2%
tertiary_educ	− 0.0286***	−0.1027	0.0861	−0.0088	32.5%	−0.0258***	− 0.0766	0.1185	− 0.0091	10.4%
unemployed	0.0200	0.0171	−0.0894	−0.0015	5.6%	0.0319***	0.0639	−0.1234	−0.0079	9.0%
inactive	−0.0107	−0.0440	− 0.0382	0.0017	−6.2%	− 0.0383***	− 0.1328	− 0.0067	0.0009	−1.0%
Ln_eqincome	− 0.0085*	− 1.1756	0.0097	− 0.0114	41.7%	− 0.0325***	−3.5180	0.0138	− 0.0486	55.5%
Residual				−0.0038	14.1%				−0.0149	17.1%
CCI				−0.0272	100.0%				−0.0875	100.0%
	2017				Total change 2007–2017					
Variables	Partial effect	Elasticity	CCI	Contribution (3)	% Contribution	Contribution (3)–(1)				
age35–64	0.0084	0.1120	0.0048	0.0005	−0.8%	− 0.0002				
age65+	−0.0049	− 0.0240	0.0032	− 0.0001	0.1%	− 0.0004				
Spanish	−0.0203**	− 0.4576	0.0052	− 0.0024	3.4%	− 0.0022				
married	0.0008	0.0108	0.0055	0.0001	−0.1%	0.0003				
separated	0.0127	0.0146	−0.0064	−0.0001	0.1%	−0.0003				
widowed	−0.0096	−0.0080	0.0034	0.0000	0.0%	−0.0001				
chronic	0.0413***	0.2672	−0.0059	−0.0016	2.2%	0.0018				
dentxinhab	0.0001	0.1799	0.0036	0.0006	−0.9%	0.0016				
lowsec_educ	−0.0044	−0.0294	− 0.0294	0.0009	−1.2%	− 0.0002				
uppersec_educ	−0.0130**	− 0.0751	0.0039	− 0.0003	0.4%	0.0008				
tertiary_educ	−0.0263***	−0.1806	0.0536	−0.0097	13.8%	−0.0009				
unemployed	0.0376***	0.1049	−0.0688	−0.0072	10.2%	−0.0057				
inactive	−0.0032	−0.0268	− 0.0027	0.0001	− 0.1%	−0.0016				
Ln_eqincome	−0.0157***	−3.6575	0.0065	−0.0238	33.9%	− 0.0125				
Residual				−0.0274	38.9%	−0.0236				
CCI				−0.0704	100.0%					

*CCI* Corrected concentration index; ***Statistically significant at 99% level (*p* < 0.01); **Statistically significant at 95% level (*p* < 0.05); *Statistically significant at 90% (*p* < 0.1)

**Table 3:** Contributions to inequality in unmet need for dental care in 2007, 2012 and 2017, and decomposition of total change. Women

	2007					2012				
Variables	Partial effect	Elasticity	CCI	Contribution (1)	% Contribution	Partial effect	Elasticity	CCI	Contribution (2)	% Contribution
age35–64	0.0213***	0.1689	0.0126	0.0021	−6.4%	− 0.0002	− 0.0012	0.0161	0.0000	0.0%
age65+	0.0009	0.0027	−0.0436	− 0.0001	0.3%	− 0.0392***	− 0.0792	− 0.0098	0.0008	− 0.8%
Spanish	− 0.0092	− 0.1356	0.0060	− 0.0008	2.4%	− 0.0363**	− 0.3434	0.0171	− 0.0059	5.8%
married	− 0.0025	− 0.0226	0.0094	− 0.0002	0.6%	0.0090	0.0521	0.0137	0.0007	−0.7%
separated	0.0214	0.0172	−0.0216	−0.0004	1.1%	0.0390**	0.0247	−0.0857	−0.0021	2.1%
widowed	0.0113	0.0192	−0.0411	−0.0008	2.4%	0.0290*	0.0326	−0.0161	−0.0005	0.5%
chronic	0.0508***	0.1999	−0.0242	−0.0048	14.5%	0.0675***	0.1821	−0.0123	−0.0022	2.2%
dentxinhab	−0.0004*	− 0.3738	0.0057	−0.0021	6.4%	0.0001	0.0940	0.0101	0.0010	−0.9%
lowsec_educ	−0.0012	− 0.0044	− 0.0334	0.0001	− 0.4%	0.0081	0.0202	−0.0655	− 0.0013	1.3%
uppersec_educ	−0.0121*	− 0.0450	0.0129	− 0.0006	1.7%	−0.0140	− 0.0337	− 0.0038	0.0001	− 0.1%
tertiary_educ	−0.0166**	− 0.0639	0.0820	−0.0052	15.7%	−0.0248**	− 0.0720	0.1211	− 0.0087	8.6%
unemployed	0.0103	0.0113	−0.0496	−0.0006	1.7%	0.0154	0.0249	−0.1008	−0.0025	2.5%
inactive	−0.0047	− 0.0364	− 0.0381	0.0014	−4.1%	− 0.0306***	−0.1440	− 0.0309	0.0045	−4.4%
Ln_eqincome	−0.0104***	−1.5968	0.0089	−0.0143	42.7%	−0.0409***	−4.1248	0.0148	−0.0609	60.2%
Residual				−0.0072	21.4%				−0.0240	23.7%
CCI				−0.0334	100.0%				−0.1012	100.0%
	2017					Total change 2007–2017				
Variables	Partial effect	Elasticity	CCI	Contribution (3)	% Contribution	Contribution (3)–(1)				
age35–64	0.0185***	0.2063	0.0092	0.0019	−2.5%	−0.0002				
age65+	−0.0033	− 0.0175	− 0.0068	0.0001	− 0.2%	0.0002				
Spanish	−0.0123	−0.2414	0.0059	−0.0014	1.8%	−0.0006				
married	−0.0111**	−0.1248	0.0117	−0.0015	1.9%	−0.0012				
separated	0.0078	0.0116	−0.0350	−0.0004	0.5%	0.0000				
widowed	0.0066	0.0183	−0.0204	−0.0004	0.5%	0.0004				
chronic	0.0442***	0.2911	−0.0123	−0.0036	4.6%	0.0012				
dentxinhab	0.0001	0.0930	0.0038	0.0004	−0.5%	0.0025				
lowsec_educ	−0.0025	−0.0126	− 0.0319	0.0004	− 0.5%	0.0003				
uppersec_educ	−0.0160***	−0.0734	− 0.0006	0.0000	− 0.1%	0.0006				
tertiary_educ	−0.0237***	−0.1499	0.0594	−0.0089	11.5%	−0.0037				
unemployed	0.0271***	0.0675	−0.0621	−0.0042	5.4%	−0.0036				
inactive	−0.0087*	−0.0852	− 0.0146	0.0012	−1.6%	− 0.0001				
Ln_eqincome	−0.0213***	−4.3222	0.0076	−0.0330	42.5%	−0.0187				
Residual				−0.0283	36.5%	−0.0212				
CCI				−0.0776	100%					

*CCI*: corrected concentration index; ***Statistically significant at 99% level (*p* < 0.01); **Statistically significant at 95% level (*p* < 0.05); *Statistically significant at 90% (*p* < 0.1)

According to Table 2, only some of the considered factors are significantly associated to the probability of men’s problems of access to dental examination or treatment for the three analyzed years. Reporting at least one chronic condition tends to increase the probability of showing unmet dental care needs, and this effect intensifies during the economic crisis. Conversely, having tertiary education, as well as income, act as protecting factors. Interestingly, the impact of income tends to grow from 2007 to 2012 and to decrease later on, although their final effect is higher at the end of the period than at the beginning of the selected decade. Moreover, some other independent variables show their influence in one or two of the selected years. This is the case of age, nationality, marital status and labour status dummies. Thereby, belonging to the 35–64 age range is positively associated with unmet needs, compared to the youngest cohorts, in the first half of the selected period. Conversely, nationality appears as a significant factor in the second half of the decade, showing those with Spanish nationality a lower probability of reporting unmet need compared to non-Spaniards. Also, marital status show significant effects in 2012, being separated, divorced and widowed more prone to reporting unmet dental care needs compared to single individuals. Unemployment arises as a significant barrier of access in 2012, which slightly intensifies its effects in 2017, despite the positive change of the economic cycle. Being inactive only shows (a negative) influence on the probability of reporting unmet needs in 2012. Finally, having upper or post-secondary non-tertiary education appears to have a significant impact on unmet needs for dental care at the beginning and at the end of the selected period, and it shows the expected sign.

The picture for women (Table 3) is similar, although some differences in coefficients arise. Particularly, nationality only appears to be relevant for females in the hardest times of the crisis. Also, unlike men, belonging to the oldest cohort significantly reduces in 2012 the probability of reporting unmet need compared to the youngest women and, interestingly, being married seems to reduce that probability compared to single females. Moreover, the effect of unemployement only appears to be significant in women at the end of the selected decade and not in the year representing the peak of the crisis. Lastly, supply of dental care, represented by the variable *dentxinhab*, seems to have a slight negative influence in the probability of reporting unmet need for dental care, but only in the 2007 model.

Positive (negative) CCIs in Tables 2 & 3 indicate a pro-rich (pro-poor) distribution of independent variables. The results show the expected sign for all the regressors, and a high similarity between men and women. However, some interesting gender differences can be observed. Firstly, the distribution of individuals over 64 years old, which changes from pro-poor to pro-rich for men along the crisis, and maintains its sign once the economy recovers, shows an invariant pro-poor bias for women. The same effect is observed for widowed. Conversely, the distribution of separated and divorced individuals, which was pro-poor before the economic crisis, becomes pro-poor for men with the crisis, whilst separated and divorced women show a pro-poor distribution along all the period. Finally, and unlike men, the distribution of upper or post-secondary education for women turns to be pro-poor with the crisis, indicating the more vulnerable position of women without university studies.

Another interesting effect is reflected in the evolution of the concentration indices for the dummy *chronic*. Although chronicity is systematically more concentrated on economically disadvantaged population, income-related inequalities in chronicity seems to have decreased along the analyzed period.

According to Tables 2 & 3, income is the factor which contributes the most to inequality for the whole period, ranging from 33.9 to 55.5% and from 42.5 to 60.2% for men and women, respectively. In both cases, the maximum contribution of income corresponds to the crisis year, being the minimum referred to the post-crisis year. Moreover, educational level appears as the second determinant of inequality due to the effect of tertiary education, ranging the total effect of the three dummies from 11.2 to 32.5% for males and from 9.8 to 17% for females. According to these results, education plays a particularly important role before the outbreak of the Great Recession and tends to reduce its importance in the crisis year, when income boosts its contribution.

Furthermore, chronicity also contributes significantly to inequality in 2007 (representing 12.5 and 14.5% for men and women, respectively). However, it loses relevance in the rest of the selected years in favor of unemployment, particularly for men, with a contribution reaching 10% at the end of the period. The share of unemployment is far more modest for women, reaching a maximum of 5.4% in 2017. One of the main differences between men and women relates to the contribution of labor status, which in the case of the latter is mainly driven by the effect of the dummy inactive. Along with unemployment, nationality also arises as a remarkable factor in 2012, being its contribution 8.2 and 5.8% for males and females, respectively.

The rest of explanatory factors shows a negligible contribution, mostly under 2%, with the exception of supply and age at the beginning of the period. Thus, the variable representing supply accounts for 3.5 and 6.4% of total income-related inequality in 2007, for men and women, respectively. Meanwhile, age explains nearly 6% in females. Lastly, the unexplained part of inequality is noteworthy in all years and tends to increase along the period, ranging from 14.1 to 38.9% for males and from 21.4 to 36.5% for females.

Tables 4 & 5 show the contribution of each factor to the evolution of inequality over the whole decade for men and women, respectively. They also provide information about if the contribution was due to changes in elasticites of the explanatory variables or to the changes in its distribution. When these changes show a negative (positive) sign, it implies that they tended to increase (decrease) inequality favouring the better-off. The final column of each table, expressed as a percentage, represents the proportion of the change in the concentration index of unmet need which is explained by each independent variable. Hence, the interpretation of signs of this last column depends on the evolution of inequality: if inequality favoring the better-off shows a rise (as it happens along the period 2007–2017), then a positive percentage indicates that the corresponding factor contributed to the increase. For the sake of simplicity, partial results for both subperiods are not shown, but they are available upon request.

**Table 4:** Oaxaca-type decomposition for total change in inequality 2007–2017. Men

	2007–2017					
	Equation (5)		Equation (6)		Total	
	*∆*CCI*Elasticity	*∆*Elasticity*CCI	*∆*CCI*Elasticity	*∆*Elasticity*CCI	Total	%
age35–64	−0.0001	−0.0001	− 0.0001	−0.0001	− 0.0002	0%
age65+	−0.0012	0.0007	−0.0004	−0.0001	− 0.0004	1%
Spanish	0.0002	−0.0024	0.0000	−0.0022	−0.0022	5%
married	0.0001	0.0002	0.0005	−0.0003	0.0003	−1%
separated	−0.0003	−0.0001	− 0.0004	0.0000	− 0.0003	1%
widowed	−0.0001	0.0000	−0.0001	0.0000	−0.0001	0%
chronic	0.0055	−0.0037	0.0026	−0.0008	0.0018	−4%
dentxinhab	−0.0006	0.0022	0.0004	0.0012	0.0016	−4%
lowsec_educ	0.0000	−0.0002	0.0000	−0.0002	−0.0002	0%
uppersec_educ	0.0009	−0.0001	0.0008	0.0000	0.0008	−2%
tertiary_educ	0.0059	−0.0067	0.0033	−0.0042	−0.0009	2%
unemployed	0.0022	−0.0078	0.0004	−0.0060	−0.0057	13%
inactive	−0.0010	−0.0007	− 0.0016	0.0000	− 0.0016	4%
Ln_eqincome	0.0115	−0.0240	0.0037	−0.0162	−0.0125	29%
Residual					−0.0236	55%
Total	0.0231	−0.0427	0.0094	−0.0290	−0.0432	100%

*CCI* Corrected concentration index

**Table 5:** Oaxaca-type decomposition for total change in inequality 2007–2017. Women

	2007–2017					
	Equation (5)		Equation (6)		Total	
	*∆*CCI*Elasticity	*∆*Elasticity*CCI	*∆*CCI*Elasticity	*∆*Elasticity*CCI	Total	%
age35–64	−0.0007	0.0005	−0.0006	0.0003	−0.0002	1%
age65+	−0.0006	0.0009	0.0001	0.0001	0.0002	−1%
Spanish	0.0000	−0.0006	0.0000	−0.0006	−0.0006	1%
married	−0.0003	−0.0010	− 0.0001	−0.0012	− 0.0012	3%
separated	−0.0002	0.0001	−0.0002	0.0002	0.0000	0%
widowed	0.0004	0.0000	0.0004	0.0000	0.0004	−1%
chronic	0.0034	−0.0022	0.0024	−0.0011	0.0012	−3%
dentxinhab	−0.0002	0.0027	0.0007	0.0018	0.0025	−6%
lowsec_educ	0.0000	0.0003	0.0000	0.0003	0.0003	−1%
uppersec_educ	0.0010	−0.0004	0.0006	0.0000	0.0006	−1%
tertiary_educ	0.0034	−0.0070	0.0014	−0.0051	−0.0037	8%
unemployed	−0.0008	−0.0028	− 0.0001	−0.0035	− 0.0036	8%
inactive	−0.0020	0.0019	−0.0009	0.0007	−0.0001	0%
Ln_eqincome	0.0057	−0.0244	0.0021	−0.0208	−0.0187	42%
Residual					−0.0212	48%
Total	0.0091	−0.0320	0.0059	−0.0289	−0.0441	100%

*CCI* Corrected concentration index

According to Table 4, income (29%) and unemployment (13%), followed by nationality (5%), were the leading factors in the evolution of inequality over the whole decade for men. These three variables contributed to increase inequalities favoring the better-off and their effect was due to changes in elasticities. Conversely, health status (chronicity) and availability of services (supply), although with a minor contribution, appear as factors that tended to reduce pro-rich inequalities. Whilst in the former case this effect was due to the reduction of income-related inequality in the distribution of chronicity, in the latter it was due to changes in elasticity.

Compared to male, the results for women (Table 5) show that, although the above mentioned variables acted in the same direction, the role of income in total change in inequality along the decade was significantly higher (42%) and that of unemployment quite lower (8%). Further, the impact of nationality seems to be negligible (1%). Also, the educational level shows a noteworthy effect (8%), so that tertiary education contributed to explain the increase of inequality favoring the better-off, again as a consequence of the variation of elasticity.

Additionally, it is worth mentioning that the contribution of the residual in the explanation of the variation of inequality between before and after the Great Recession reaches 55% for males and 48% for women. Thus, nearly half of the evolution of inequality remains unexplained.

## Discussion

The results show a significant income-related inequality favouring the better-off in the distribution of unmet need for dental care in Spain all over the decade 2007–2017, which is consistent with the available evidence for some years of the same period [25, 31, 32], and also with the fact that most dental care for adult Spaniards is excluded from public coverage.

Furthermore, a pro-cyclical behavior of inequality of unmet need has been found: during the Great Recession inequality tended to rise, in line with previous evidence [32], while it tended to decline as the economic recovery started. Although the proportion of people reporting unmet needs registered a positive trend along the analyzed decade, the CCIs show that inequality increased in Spain over time, as well as it happened to income distribution, as revealed by the evolution of the Gini index [14].

The probability of reporting unmet need for dental care was mostly (and negatively) associated with income and educational level for the whole period, which is consistent with available evidence [10, 11, 27, 30–35, 46]. Conversely, people with health problems (proxied by chronicity), non-Spaniards and unemployed were more likely to report unmet needs, also in line with previous results [10, 11, 27, 30–32]. As it may also be seen in Table S1 (supplementary file), those population groups showed high prevalence of unmet needs, particularly in the hardest part of the economic crisis. Some significant differences between men and women were detected, particularly regarding the influence of income (more intense in women’s models) and unemployment (more important in men’s models), a phenomenon that had been shown previously [32]. Furthermore, the slight influence of dental care supply (only significant at the beginning of the period and only for women), is consistent with the low percentage of population reporting unmet need as a consequence of waiting list, services being too far or lacking references about good professionals [9].

The results of probit models allow the identification of most vulnerable groups: migrants, unemployed, chronically ill, uneducated and low-income people, who should be on the focus of policy making in order to combat unmet dental care needs. With the economic crisis, their vulnerability tended to rise and also new vulnerable groups appeared, such as those being separated, divorced or widowed, suggesting the relevance of family support during hardest times. The particular influence of income and unemployement during the Great Recession had been previously shown for Spain [32] and for other European countries [11]. However, the results here presented prove that the influence of those factors persisted after the end of the Recession, and that their impact in the post-crisis period is significantly higher than before the crisis.

Regarding the distribution of variables, it was found that the distribution of males over 64 years old -and also of widowed- became pro-rich with the crisis, while keeping an invariant pro-poor bias for women. This may be due, firstly, to the relative rigidity of old age pension benefits compared to the visible decline of market incomes for younger groups [47], since young Spaniards were hardly hit by unemployment when the Great Recession started (and they still are). Secondly, it could be related to the high differences in the average amount of public benefits for men and women, being the latter nearly 40% lower than the former [48]. Also, the results revealed some decline in income-related inequalities in chronicity along the analyzed decade, mainly for men, which is consistent with previous evidence [44] and partially explained by the same reason: the relative protection of pensioners’ income [44, 47].

Moreover, the analysis shows that income was the main determinant of inequality and of its variation along time -particularly for women-, which would be related to the absence of universal dental care insurance coverage [46]. The higher role of income in unmet need along time is also reflected in the evolution of the proportion of people declaring that the main reason for not receiving dental care is that they cannot afford its cost, along the analyzed decade: from 41.17 to 90.13% for men, and from 51.81 to 91.27% for women [9]. Also, the results obtained indicate that the role played by income in the evolution of inequality is mainly due to the changes in elasticities. The same effect is seen with respect to unemployment, although its impact on inequality change is far less important. As it has been suggested by some authors [32, 49], in a context of economic concern consumers tend to forego dental care in order to satisfy other priority needs. Unfortunately, and despite the official end of the Great Recession, the Spanish labor market still shows structural challenges such as high unemployment and temporary employment rates, and also low salaries. These results, once again, point to the role played by social determinants in access inequalities to dental/health care and, hence, in health inequalities. Therefore, public policies aimed at improving living and labor conditions should be a priority for policy makers in order to reduce health inequities.

Otherwise, the contribution of nationality to the increase of pro-rich inequality has been quite modest along the analyzed decade. Although the particularly vulnerable situation of migrants during the Great Recession was reflected by the estimated models, the revocation of full right to public health care coverage for undocumented migrants in 2012 has not played any important role in the evolution of inequality, since dental care is mostly excluded from the public basket of benefits.

Moreover, the estimated residuals both in the probit models and in the decomposition of income-related inequality change are significant. This may be related to the limited number of explanatory factors considered in the models and to the absence of objective indicators of dental care need, which has been conditioned by their availability in the EU-SILC. It has to be taken into account that this data source is not specifically designed to analyze neither health care nor dental care access. Further, when the dependent variable takes value of 0 two different interpretations are possible: first, that the individual perceived the need of visiting a dentist but didn’t satisfy it or, alternatively, that he/she didn’t perceive the need of dental care. The distinction between both categories would be very useful in order to better identify the determinants of unmet dental care needs, but it is not possible for the period prior to 2015. Only since 2015 the question used to retrieve information about unmet need was modified by dividing the original question into the following two: “*Was there any time during the last 12 months when you personally needed a dental examination or treatment?”* and *“Have you received that care every time you needed it?”*. Other Spanish databases also collect information about unmet dental needs, such as the Spanish Health Interview Survey, but it only does in its latest edition. Besides, the choice of the data source is justified by the convenience of having a precise income variable in order to compute concentration indices.

Furthermore, the magnitude of residuals is consistent with previous studies analyzing changes in health and health care inequalities during the Great Recession, which suggest that changes over the economic crisis may be partially explained by factors and trends that are not captured by survey data [50, 51]. Additionally, the high contribution of unexplained factors could be related to the difficulty to properly identify the determinants of dental care needs [52].

Lastly, as Coveney et al. (2020) suggest, the change in the concentration indices in Spain may mask the existence of two underlying drivers: changes in income ranks and changes of the dependent variable over time [47]. Thus, disentangling both effects would be interesting. Unfortunately, the EU-SILC is set up as a rotating panel where the sample is completely renewed every four years. Since this is a cross-sectional study, causal interpretation of the findings is not possible. But despite this caveat, and as stated by Pulok et al. (2020), the measurement of changes in inequality over time may be helpful to inform policy decision-making [53].

## Conclusions

The scheme of dental care in Spain, mainly provided and financed by the private sector, leads to income-related inequalities in unmet needs and, by extension, in oral health. Far from decreasing in recent years, inequality in unmet dental care needs tended to grow. Thus, the Great Recession left its trace in form of a higher inequality in the access to dental care. Another visible trace would be that unmet need for dental care, as well as its inequality, became more sensitive to ability to pay and to unemployment. Before Spaniards could have forgotten the effects of the Great Recession, a new (and probably harder) crisis has come as a consequence of the COVID-19 pandemics. If public authorities want to prevent inequalities from furtherly increasing, some actions should be developed. In this sense, to broaden public coverage of dental care for vulnerable groups, such as low-income/unemployed people with high oral health needs, would help to reduce the social gradient in barriers of access. Also, as it has been suggested elsewhere [54], addressing the underlying causes of inequalities in oral health would reduce the dependence of dental treatments and thus would contribute to oral health equity.

## Supplementary Information

**Additional file 1.**

## Data Availability

The datasets supporting the conclusions of this article are available upon request to Eurostat. To apply for access consult: https://ec.europa.eu/eurostat/documents/203647/771732/How_to_apply_for_microdata_access.pdf
