# Supplementary material for: Income-related inequalities in unmet dental care needs in Spain: traces left by the Great Recession
Source: Int J Equity Health. 2020 Nov 12;19:207. doi: 10.1186/s12939-020-01317-x (PMC7658913; doi:10.1186/s12939-020-01317-x)
Supplement: Supplementary file 1 — Additional file 1. [file 12939_2020_1317_MOESM1_ESM.docx]

Table S1. Unmet needs by characteristics of the sample.

|  | Mean 2007 | | Mean 2012 | | Mean 2017 | |
| --- | --- | --- | --- | --- | --- | --- |
| Variables | Men | Women | Men | Women | Men | Women |
| age16-34 | 5.1% | 4.1% | 6.9% | 9.3% | 3.1% | 3.1% |
| age35-64 | 8.3% | 7.2% | 10.7% | 10.5% | 4.8% | 5.6% |
| age65+ | 6.5% | 7.0% | 6.3% | 6.7% | 3.3% | 4.2% |
| Spanish | 6.9% | 6.2% | 8.0% | 8.8% | 3.8% | 4.4% |
| non-Spanish | 6.9% | 5.9% | 15.2% | 15.0% | 7.5% | 7.2% |
| single | 5.6% | 4.7% | 7.3% | 8.6% | 3.7% | 4.2% |
| married | 7.6% | 6.2% | 9.4% | 9.2% | 4.2% | 3.9% |
| separated | 11.0% | 9.9% | 13.9% | 16.2% | 6.9% | 9.0% |
| widowed | 5.2% | 8.4% | 8.9% | 9.0% | 2.8% | 6.5% |
| chronic | 10.3% | 10.7% | 12.3% | 13.2% | 7.5% | 8.3% |
| non-chronic | 5.9% | 4.7% | 7.8% | 8.2% | 2.9% | 3.0% |
| primary_educ | 9.0% | 7.9% | 10.3% | 10.0% | 6.3% | 6.5% |
| lowsec_educ | 7.3% | 7.0% | 10.5% | 12.1% | 5.5% | 6.5% |
| uppersec_educ | 5.9% | 4.9% | 8.9% | 9.4% | 3.5% | 3.7% |
| tertiary_educ | 4.9% | 4.2% | 5.6% | 6.6% | 1.5% | 2.2% |
| working | 6.7% | 5.3% | 8.3% | 9.0% | 2.9% | 3.5% |
| unemployed | 10.2% | 7.6% | 15.3% | 14.9% | 11.1% | 10.7% |
| inactive | 6.5% | 6.7% | 6.0% | 8.0% | 3.6% | 4.3% |
| Eqincome (Q1) | 10.2% | 8.8% | 14.7% | 14.6% | 9.9% | 10.3% |
| Eqincome (Q2) | 7.5% | 6.5% | 10.8% | 12.9% | 5.0% | 6.1% |
| Eqincome (Q3) | 6.3% | 5.9% | 8.4% | 8.9% | 2.9% | 3.9% |
| Eqincome (Q4) | 6.7% | 5.7% | 7.1% | 7.1% | 1.8% | 2.0% |
| Eqincome (Q5) | 4.5% | 3.7% | 4.1% | 4.1% | 0.9% | 1.0% |

Q1-Q5: equivalent income quintiles from the poorest (Q1) to the richest (Q5).
